# Monitoring Chemical Environmental Hazards Through Wildlife Assessment: A Review Within the “One Health” Approach

**DOI:** 10.3390/jox16020057

**Published:** 2026-03-25

**Authors:** Claudia A. Rocha, Luís M. Félix, Dércia Santos, Sandra M. Monteiro, Carlos Venâncio

**Affiliations:** 1 Department of Animal Science, School of Agrarian and Veterinary Sciences (ECAV), University of Trás-Os-Montes and Alto Douro (UTAD), Quinta de Prados, 5000-801 Vila Real, Portugal; xana3@live.com.pt; 2 Department of Biology and Environment, School of Life and Environmental Sciences (ECVA), University of Trás-Os-Montes and Alto Douro (UTAD), Quinta de Prados, 5000-801 Vila Real, Portugal; lfelix@utad.pt (L.M.F.); dsantos@utad.pt (D.S.); smonteir@utad.pt (S.M.M.); 3 Centre for the Research and Technology of Agroenvironmental and Biological Sciences (CITAB), Inov4Agro, University of Trás-Os-Montes and Alto Douro (UTAD), Quinta de Prados, 5000-801 Vila Real, Portugal

**Keywords:** contaminants, food safety, game meat, metals, microplastics

## Abstract

Wildlife acts as a sentinel of environmental pollution, providing critical insights into potential risks to human health within the *One Health* framework. However, knowledge on the occurrence of legacy and emerging contaminants in wildlife, as well as their potential trophic transfer to humans, remains limited. Thus, monitoring contaminants in terrestrial wildlife, particularly in game species, is especially relevant, as game meat represents an important source of high-quality protein that must be safeguarded. This review summarizes current evidence on chemical contaminant levels in terrestrial wildlife from a “One Health” perspective. Despite the growing relevance of this approach, few studies have explicitly applied this term, and even fewer have focused on game meat, resulting in an incomplete picture of contamination. Although reported contaminants—metals, metalloids, pesticides, microplastics, and mycotoxins—originate from overlapping natural and anthropogenic sources, such as ammunition, agriculture, and industrial activities, a strong dependence on local environmental conditions continues to hamper cross-regional comparisons and the establishment of representative exposure levels. Overall, this review highlights the need for systematic monitoring of contaminants in terrestrial wildlife, with emphasis on emerging pollutants that are currently underrepresented in literature, to improve risk assessment, protect food safety, and better understand the impacts of environmental contamination on animal and human health.

## 1. Introduction

The increase in human population, industrialization, intensive agriculture, and other anthropogenic activities has caused environmental degradation, negatively affecting ecosystems and biodiversity through the continuous release of environmental contaminants [1,2,3]. Once released, these pollutants persist in soil, water, and air, facilitating their uptake and accumulation in wild animals [4,5,6]. Consequently, the consumption of wildlife and game meat represents an important route of human exposure, promoting bioaccumulation and bioamplification along the food chain [4,5]. This scenario is particularly relevant for remote populations that depend on wildlife for nutrition and therefore have an increased risk of contaminant exposure [7,8]. In this context, wild fauna serves a dual purpose as it can act as a sentinel of ecosystem contamination while also identifying potential risks to human health through game meat consumption. By reflecting both environmental qualities and potential dietary risks, wildlife monitoring provides an important tool that connects ecosystem health, food safety, and human well-being, further reinforcing the relevance of an integrated monitoring approaches and the importance of adopting the “One Health” concept.

Despite increasing awareness of environmental pollution, most studies addressing contaminant exposure on wildlife and game meat have mainly focused on a limited group of legacy contaminants, particularly metals, due to their persistence, bioaccumulative nature, and well-documented toxicological effects [9,10,11,12]. However, this narrow focus has resulted in a significant knowledge gap regarding other emerging contaminants, such as persistent organic pollutants, pesticides, mycotoxins, and microplastics [12,13,14,15,16,17]. These substances originate from diverse anthropogenic sources, such as unregulated agricultural practices, industrial discharges, and improper waste management, entering the ecosystems and accumulating in wildlife tissues and food webs, consequently exposing human consumers of game meat to emerging contaminants with a range of potential undesirable health effects [17]. The occurrence of contaminant-related diseases and sub-lethal dysfunctions in wildlife highlights the complex interconnections between environmental integrity, animal, and human health, ultimately accentuating the relevance of the “One Health” framework [2,12]. This concept recognizes that human health is inseparable from animal health and the ecosystems they inhabit and emphasizes the need for having integrated and multidisciplinary approaches to risk assessment and environmental monitoring [12].

In this context, game meat constitutes an important source of high-quality protein that should be valued and safeguarded, especially in rural and hunting-dependent communities [18,19]. Therefore, ensuring its safety is essential. Yet, regulations governing chemical contaminants in game meat remain inconsistent across countries, and contaminant levels are often assessed using reference levels established for farmed animals which are often raised under controlled conditions and management practices and thus do not accurately reflect environmental exposure [11,18]. Contrastingly, the free-roaming nature of wildlife results in site-specific contaminant exposure that makes it a more sensitive and ecologically relevant indicator of ecosystem contamination [16]. Consequently, this regulatory mismatch hampers accurate risk assessment and complicates comparisons across studies and regions.

Given these limitations and the more direct link between terrestrial ecosystems, wildlife and human exposure pathways, a comprehensive understanding of how chemical environmental contaminants affect terrestrial wild animals and how they may reach humans through game meat is essential. This review aims to compile and critically analyze current evidence on chemical contaminants of concern in terrestrial wildlife and game meat while examining how the “One Health” concept is applied, identifying major literature gaps and highlighting priorities for future research and monitoring strategies. Although aquatic fauna also represents a set of important sentinels of environmental contamination, this review intentionally focuses on terrestrial systems to enable a more targeted synthesis of evidence.

## 2. Materials and Methods

### 2.1. Search Strategy and Eligibility Criteria

To emphasize the importance of investigating environmental contaminants and the dual role of wildlife species as both sentinels and vectors, a literature search was conducted in SCOPUS, as this database reports the highest number of indexed documents [20]. The Boolean connector “AND” was used to combine keywords related to game meat and wildlife to those related to environmental contaminants. The connector “OR” was applied within these groups as follows: (“game meat” OR wild” OR “wildlife”) AND (“heavy metals” OR “chemical contaminants” OR “emerging organic pollutants” OR “emerging organic contaminants” OR “lead” OR “mercury” OR “nickel” OR “arsenic” OR “cadmium” OR “polychlorinated” OR “pesticides” OR “radionuclides” OR “radioactive elements” OR “mycotoxins” OR “dioxins” OR “polycyclic aromatic hydrocarbons” OR “phthalate” OR “antimicrobials” OR “plastics” OR “psychoactive substance”).

The studies considered for this review were novel, non-retracted, peer reviewed research papers published before February 2026, that examined one or more contaminants in game meat and included the term “One Health”. Non-English papers, reviews, abstracts or books, as well as studies focusing on divergent topics, such as microbiology, zoonoses, or aquatic fauna were excluded.

### 2.2. Screening and Data Organization

Following the database search for the selected keywords, a filter (filter 1) was applied to remove studies not explicitly mentioning the “One Health” concept, aiming to specifically assess how this framework is currently applied in terrestrial wildlife contaminant monitoring. An additional filter was applied to ensure relevance to chemical contaminants (filter 2) using the keywords “pathogen”, “spillover”, “resistance”, “maldi-tof”, “genomic”, “infection”, “transmission”, “bacteria”, “virus”, “COVID-19”, “reform”, “parasitology”, and “fascioloides”. The remaining studies were filtered (filter 3) using the keywords “seawaters”, “seabirds”, and “fishes”, to eliminate water-related data and maintain focus on terrestrial game species.

The final search results were screened based on title and abstract and according to pre-established inclusion and exclusion criteria. The selected papers were organized in an Excel sheet, and data on the year of publication, study location, species, evaluated contaminants and their respective concentration (mean and standard deviation (SD)) and sample matrix were extracted. To ensure consistency across the dataset, SD was calculated from the standard error of the mean (SE) when necessary, and units were standardized to a uniform format whenever possible. When data was presented solely in graph format, values were extracted using WebPlot Digitizer (Version 4.8) for analysis and mean and SD were calculated according to the method described by Wan et al. (2014) [21].

Three authors (C.A.R., D.S., and C.V.) screened the retrieved literature and cross-checked the data according to the inclusion and exclusion criteria. Any questions or uncertainties were resolved with the assistance of another author (LF, or SMM).

## 3. Results

### 3.1. Study Selection and Screening Results

**Figure 1:**
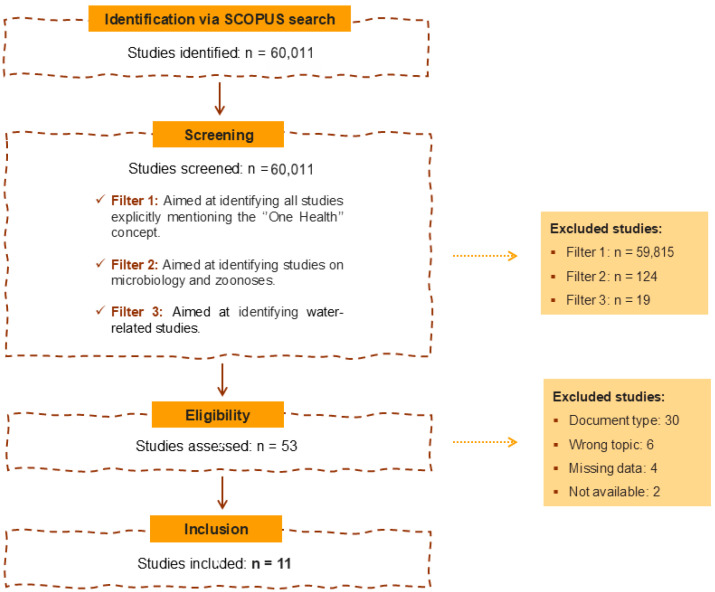
Workflow performed to screen studies for this review, indicating the reasons for exclusion and the number of scientific articles in each phase.

A total of 60,011 papers assessing environmental contaminants on wildlife were initially retrieved from the SCOPUS database (Figure 1). Following the first filter, screening for the presence of the term “One Health”, 196 studies remained. Of these, 124 records were excluded, leaving 72 studies for the next filtering step. In the third filtering stage, 19 papers were excluded, resulting in 26 eligible papers. Finally, 11 studies met all inclusion criteria and were retained for qualitative analysis.

### 3.2. General Characteristics of the Included Studies

**Figure 2:**
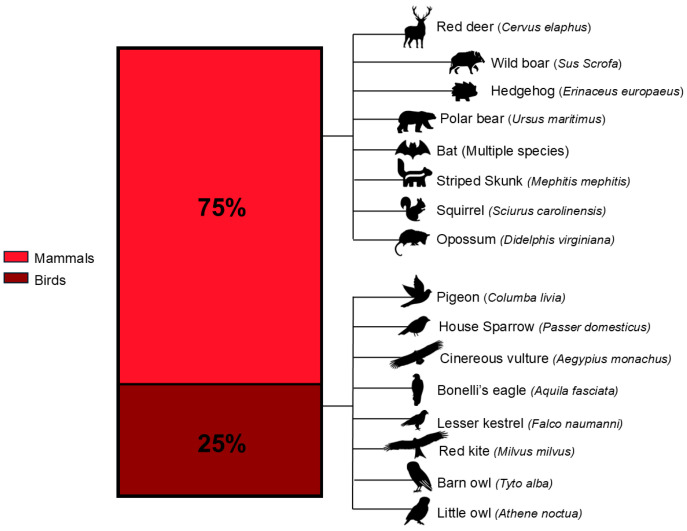
Distribution of the 11 eligible studies by taxonomic category (mammals and birds), with species positioned farther from the central bar reported in two studies each and species closer reported in a single study. Since some studies mentioned both mammals and birds, percentages were calculated based on the total number of mentions (*n* = 12). The investigated bat species included the Fulvous fruit bat (*Rousettus leschenaultia*), the Great roundleaf bat (*Hipposideros armiger*), the Chinese rufous horseshoe bat (*Rhinolophus sinicus*), the Large myotis (*Myotis chinensis*), the Great evening bat (*Ia io*), the Eastern bet-wing bat (*Miniopterus fuliginosus*), the Black bearded tomb bat (*Taphozous melanopogon*). The graph was created using GraphPad Prism 9 for Windows (Version 9.5.0; La Jolla, CA, USA).

The final dataset comprised 11 recently published studies, with four articles from 2023, four from 2024, two from 2025, and one from 2026, reflecting the emerging nature of this research topic. Most studies focused on mammalian species (75%), whereas avian species accounted for 25% of the investigated fauna (Figure 2). While the majority of the species were addressed in a single study, wild boar (*Sus scrofa*) and hedgehog (*Erinaceus europaeus*) were each investigated in two independent studies.

### 3.3. Species and Geographic Distribution

Mammalian species include red deer (*Cervus elaphus*) [22], wild boar (*Sus scrofa*) [15,23], hedgehog (*Erinaceus europaeus*) [24,25], polar bear (*Ursus maritimus*) [26], multiple bat species, such as the Fulvous fruit bat (*Rousettus leschenaultia*), the Great roundleaf bat (*Hipposideros armiger*), the Chinese rufous horseshoe bat (*Rhinolophus sinicus*), the Large myotis (*Myotis chinensis*), the Great evening bat (*Ia io*), the Eastern bet-wing bat (*Miniopterus fuliginosus*), and the Black bearded tomb bat (*Taphozous melanopogon*) [27], striped skunk (*Mephitis mephitis*) [28], squirrel (*Sciurus carolinensis*) [29], and opossum (*Didelphis virginiana*) [29]. Avian species comprised pigeons (*Columba livia*) [24], house sparrows (*Passer domesticus*) [30,31], and raptor species, among which were the Cinereous vulture (*Aegypius monachus*), the Bonelli’s eagle (*Aquila fasciata*), the Lesser Kestrel (*Falco naumanni*), the Red Kite (*Milvus milvus*), the Barn Owl (*Tyto alba*), the Little owl (*Athene noctua*) [32].

Geographically, three studies were conducted in Portugal, and included samples from wildlife centres in the north (CERVAS-ALDEIA—Centre of Ecology, Wild animals’ Rehabilitation and Surveillance, in Guarda), centre (LxCRAS—Lisbon Wildlife Rescue centre, in Lisbon), and south (RIAS-ALDEIA—Wildlife Rehabilitation and Research Centre, in Olhão) [24,25], as well as from Lousã and Idanha-a-Nova [22]. Two studies were conducted in Spain, covering regions such as Castile and Léon [23], and multiple provinces across the mainland (Almería, Granada, Cádiz, Córdoba, Jaén, Málaga, Madrid, Ávila, Burgos, La Rioja, Salamanca, Cáceres, Zamora, and Badajoz) and the Balearic Islands (Mallorca) [32], and another two in the United States of America (Minesota [29] and Michigan [28]). Additional studies were conducted in Australia, with sampling sites in New South Wales (Broken Hill, Cobar, Dubbo, and Richmond) and Queensland (Mount Isa, Cloncurry, and Townsville) [31], Italy (Avellino province) [15], Arctic Canada (Northern Beaufort Sea, Southern Hudson Bay, Western Hudson Bay, Baffin Bay, Foxe Basin, and Gulf of Boothia) [26], and in China (Yunnan Province) [27]. When available, contaminant levels were reported separately for each region within a country.

### 3.4. Types of Contaminants Assessed

**Figure 3:**
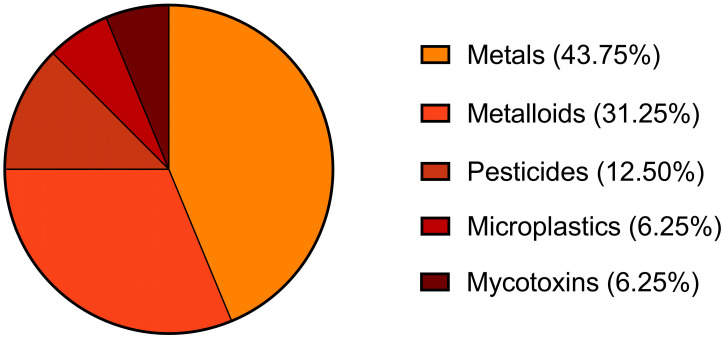
Contaminants assessed in the 11 eligible studies. Since some studies mentioned more than one pollutant, percentages were calculated based on the total number of mentions (n = 16). The graph was created using GraphPad Prism 9 for Windows (Version 9.5.0; La Jolla, CA, USA).

The contaminants investigated across the selected studies (Figure 3) were predominantly metals (43.75%), such as lead (Pb) [19,20,23,24,25,26], cadmium (Cd) [19,20,21,22,23], mercury (Hg) [23], and nickel (Ni) [20]. Metalloids accounted 31.25%, represented exclusively by arsenic (Ar) [19,20,21,22,23]. Emerging contaminants were less frequently addressed, with pesticides, among which are organochlorine insecticides, including chlordane and its metabolites (oxychlordane, heptachlor epoxide, and trans-nonachlor) [28], organophosphorus insecticides, carbamate insecticides, pyrethroid insecticides, acaricides, herbicides, fungicides, as well as other categories [27], representing 12.50% of the analysed contaminants. Microplastics [32] and mycotoxins, namely zearalenone (ZEN) and its metabolite α-zearalenol (α-ZEL), represent a smaller fraction (6.25%) of the literature [15].

Several studies also reported additional elements, such as zinc (Zn), strontium (Sr), sulphur (S), phosphorous (P), sodium (Na), molybdenum (Mo), manganese (Mn), magnesium (Mg), potassium (K), calcium (Ca), barium (Ba), silver (Ag), iron (Fe), cobalt (Co), chromium (Cr), and copper (Cu). However, these were not considered within the scope of this review, as they are either essential trace elements or lack well-established toxicological thresholds, limiting their interpretation in a contamination risk context [22,23,25,26,33]. In contrast, arsenic (As) and nickel (Ni) were retained given their established association with polluted environments and recognised ecotoxicological relevance [23,25,33].

### 3.5. Contaminant Concentration Across Species

The highest concentrations of lead (449.55 ± 3144.01 mg/kg dry weight), nickel (0.36 ± 0.69 mg/kg dry weight) and arsenic (2.50 ± 3.29 mg/kg dry weight) were reported in faecal samples from polar bears inhabiting Arctic regions [26] (Table 1). The highest cadmium concentration (13.06 ± 9.10 mg/kg dry weight) was detected in the kidneys of red deer from Lousã [22]. Lead was also quantified in blood, with the highest levels observed in house sparrows (31.4 ± 21.1 mg/dL) [31]. Mercury was exclusively analysed in polar bears, with peak liver concentrations of methylmercury and total mercury reaching 2.37 ± 2.50 and 45.86 ± 41.89 mg/kg dry weight, respectively [26]. The highest concentrations of organochlorine insecticides, specifically chlordane and its metabolites, were reached in brain (4500 ± 7120 ng/g wet weight), and liver (87,200 ± 201,000 ng/g wet weight) of Striped skunks, consistent with the tissue’s lipid content (brain: 101,000 ± 110,000 ng/g lipid weight; liver: 697,000 ± 1,030,000 ng/g lipid weight) (Table 2) [28]. The fulvous fruit bat revealed the highest concentrations of organophosphorus insecticides (1149.19 ± 1648.08 µg/kg), carbamate insecticides (160.27 ± 182.07 µg/kg), pyrethroid insecticides (156.14 ± 192.06 µg/kg), herbicides (1962.71 ± 3719.72 µg/kg), acaricides (412.66 ± 754.38 µg/kg), fungicides (1297.33 ± 1965.89 µg/kg), and others (123.17 ± 231.24 µg/kg) [27]. Microplastics, categorised as artificial fibers (AFs) and microparticles (MPs), showed the highest accumulation in regurgitated pellets, particularly from the barn owl (7.90 ± 3.97 AFs/pellet) and red kite (4.21 ± 0.95 MPs/pellet), (Table 3) [32]. Regarding mycotoxins, the highest concentration of ZEN (1.71 ± 1.98 ng/g) was detected in the liver and α-ZEL (0.77 ± 0.98 ng/g) in the kidney of wild boars, the only species evaluated for these compounds (Table 4) [15].

## 4. Discussion

Human activities are major drivers of environmental contamination through the release of chemical pollutants into air, water, and soil [1,2,3,6]. Once introduced into the environment, these contaminants can be absorbed, distributed, and accumulated by wildlife, ultimately entering the human food chain through the consumption of game meat or other animal-derived products, and posing potential risks to human health [4,9]. Within this context, the “One Health” concept highlights the intrinsic connection between environmental integrity, animal health, and human well-being, advocating for integrated and multidisciplinary approaches to risk assessment and mitigation [2,34]. By explicitly adopting a *One Health* perspective, this review aimed to identify and synthesize evidence linking chemical contamination in wildlife to potential implications for ecosystem and human health.

The present review was restricted to studies explicitly referring to the “One Health” concept, aiming to assess how this framework is currently applied in terrestrial wildlife contaminant monitoring. As a result, relevant studies addressing similar issues without using this terminology may have been excluded, representing an important methodological limitation. Nevertheless, this accentuates the inconsistent adoption of the “One Health” terminology in the literature, which is clearly illustrated by the substantial discrepancy between the SCOPUS search results (60,011) and the ones excluded after the first screening step (59,815), of which only eleven met the inclusion criteria. In addition, since studies focusing on zoonoses were eliminated—and considering the significant relevance of this topic within the *One Health* concept—it is possible that relevant studies addressing both zoonotic agents and contaminants of interest to this review were also excluded, representing a further methodological limitation. Furthermore, the literature search was restricted to English papers and relevant papers in other languages may have been excluded, potentially introducing language bias and contributing to the underrepresentation of some regions. Among the included studies, three focused on game meat from red deer (*Cervus elaphus*) and wild boar (*Sus scrofa*) across Italy [15], Portugal [22], and Spain [23]. The limited number of game species, together with the restricted taxonomic coverage of wildlife, provides only a partial picture of contamination patterns. In addition, contaminant accumulation varied markedly among species, organs, and geographic regions, reflecting different local environmental conditions and exposure pathways, which complicates cross-regional comparisons and the definition of reference concentrations. Furthermore, only three studies assessed the impact of contaminant exposure on animal populations, revealing an important gap to address in future studies. Nevertheless, the reported observations remain relevant, as clear histopathological alterations were observed in the livers and kidneys of red deer [22] and wild boar [23], as well as in the liver of hedgehogs [25], potentially suggesting a link between contaminants and these alterations.

Notably, polar bears exhibited the highest concentrations of lead, nickel, and arsenic in faecal samples, suggesting a substantial contamination burden in the Arctic region and accentuating the strong potential for bioamplification of these pollutants along Arctic food webs, where polar bears occupy the apex predator niche [26,35]. High lead concentrations in apex predators often reflect widespread contamination across lower trophic levels, with potential health effects on numerous other wildlife species beyond polar bears that must not be overlooked. Moreover, although consumption of polar bear meat is largely restricted to Inuit communities, this dietary exposure pathway represents a clear “One Health” concern that reflects the broader risk associated with the consumption of apex predators’ meat worldwide and therefore warrants attention. High contaminant levels are also observed in herbivores, such as cadmium in the kidneys of red deer [22], as well as in omnivores, such as the house sparrows, where blood lead was elevated [31]. Similarly, multiple classes of pesticides were reported at high concentrations in the fulvous fruit bats [27], while Afs and MPs were prevalent in barn owls and red kites, respectively [32]. While these findings illustrate that high contaminant burdens may affect different species and trophic levels, the difficulty of establishing comparisons across substances is also noted, as some contaminants were only investigated in a single species, underscoring the need for broader taxonomic monitoring to better understand their ecological and health risks.

Lead was the most extensively investigated contaminant across the reviewed studies. This non-biodegradable metal occurs naturally at low concentrations but is widely introduced into the environment through anthropogenic activities, such as mining, industrial processes, and hunting practices [9,22,36,37]. Lead-based ammunition represents a major source of contamination in game meat, as bullet fragments can disperse within animal tissues during hunting [9,36]. Consequently, consumption of contaminated meat is a significant route of human exposure, especially given that no safe blood lead concentration has been established [5,36]. Nevertheless, while lead was detected in several tissues of game species assessed here, the highest accumulation levels were observed in red deer from Idanha-a-Nova, a pattern justified by the proximity to a mining area rather than hunting activity [22]. This finding underscores the presence of multiple exposure pathways beyond ammunition-derived sources, including contaminated water and forage, to which scavenger species such as raptors are especially vulnerable due to their ingestion of contaminated carcasses [9,38,39,40].

Cadmium is a rare element released to the environment through the weathering of rocks and volcanic activity, although its higher concentrations are typically associated with human activities, such as mining, industrial processes, and the excessive use of chemical fertilizers [10,41]. As a result, cadmium pollution tends to be more common in heavily industrialized areas where the water, air, and soil are more likely to be affected [33]. Cadmium contamination was consistently reported across species, with the highest values detected in kidneys of red deer from Portugal [22,24]. These tissue-specific accumulation patterns suggest chronic exposure, likely linked to dietary intake, as cadmium readily accumulates in plant roots and leaves consumed by herbivores and omnivorous species [22,24,25,26,38]. These findings raise concerns for human consumers of game meat, given cadmium’s well-established nephrotoxic and carcinogenic properties [22,42].

Arsenic, a recognized carcinogenic metalloid, was detected across multiple species and regions [22,23,24,25,26]. Although it occurs naturally, its environmental presence is significantly amplified by mining, smelting, glass manufacturing, and pesticide use, leading to water reservoirs and food webs [9,22,23,24,25,26,39]. Chronic exposure has been linked to cancer, cardiovascular disease, and neurotoxicity, reinforcing its relevance within a One Health framework [43].

Despite their recognized toxicity, nickel and mercury were assessed in only one study each [26], revealing a significant gap in wildlife monitoring. Given the genotoxic and bioaccumulative properties of these elements, broader and more systematic surveillance across taxa and regions is urgently needed [9,26,44,45,46].

Emerging contaminants are natural, synthetic, or biological substances that are detected in the environment and represent an increasing concern under the “One Health” paradigm [17]. Despite their ecological relevance, only four studies on these contaminants met the inclusion criteria of this review, reinforcing the need for further research that explicitly integrates them into a “One Health” monitoring framework [16].

Pesticides are extensively used in agriculture to improve productivity and are subject to strict regulations [16,47,48]. Nevertheless, misuse and illegal application still occur and remain important drivers of wildlife exposure and contamination [47]. These types of contaminants can cause a wide range of adverse effects, including neurotoxicity and hormone-disruption effects, on humans and other non-target organisms, ultimately affecting biodiversity and ecosystem equilibrium and outweighing the intended benefits of plant protection [16,49].

Microplastics, now ubiquitous across terrestrial and aquatic ecosystems, were addressed in only one of the reviewed studies [17,32]. In addition to their intrinsic toxicity, microplastics can act as vectors for other pollutants, including metals and organic compounds, thereby amplifying exposure risks for wildlife and humans [14,50]. The scarcity of data on microplastics in terrestrial wildlife highlights a crucial research gap, particularly considering the rapid increase in plastic production and environmental dissemination [17].

Similarly, mycotoxins are largely understudied in wildlife and game meat, with only one study reporting the presence of ZEN and its metabolite, α-ZEL, in wild boar tissues [15]. Mycotoxins are toxic secondary metabolites produced by filamentous fungi that can enter the food chain through contaminated plants, meat, or other animal-derived products [51]. As fungal growth and mycotoxin production are strongly influenced by climatic conditions, climate change is expected to exacerbate contamination risks in terrestrial food webs [51]. Thus, monitoring of mycotoxins in wildlife is essential for anticipating future risks to the ecosystem and human health [15,51].

This review reveals substantial gaps in the monitoring of both legacy and emerging contaminants in wildlife within the “One Health” context. While metals, such as lead and cadmium, are relatively well documented, other contaminants of comparable concern, including mercury, nickel, pesticides, microplastics, and mycotoxins, remain severely underrepresented. This imbalance limits comprehensive risk assessment and hinders the development of effective mitigation strategies.

## 5. Conclusions

Several chemical environmental contaminants, including metals, metalloids, pesticides, microplastics, and mycotoxins across Europe (Italy, Portugal, Spain), Asia (China), America (United States of America) and Oceania (Australia) were addressed in this review. Besides the clear discrepancy between legacy and emerging contaminants, some metals are more thoroughly investigated than others. In addition, some methodological limitations must also be considered, as this review (1) only included studies explicitly mentioning the “One Health” term, (2) excluded zoonotic-related research, and (3) eliminated non-English papers, which may have resulted in the loss of relevant information. Despite this, these findings still reveal important gaps regarding taxonomic range and geographic representation, which, alongside the narrow contaminant focus, result in an incomplete picture of wildlife contamination.

Overall, these results emphasize the urgent need for harmonized and comprehensive monitoring strategies that explicitly adopt a One Health framework, expanding the scope to include underrepresented but highly relevant emerging pollutants to better protect ecosystem integrity, wildlife health, and human well-being. In the future, it is important to strengthen environmental surveillance by monitoring clinical signs alongside chemical presence in tissues, aiming to improve risk assessment, protect food safety, and better understand the impacts of environmental contamination on wildlife and human health.

## Figures and Tables

**Table 1:** Metal and metalloid accumulation levels (median ± SD) detected across different species, sampling matrices, and geographic locations.

	Study	Country	Region	Species	Sample	Contaminant	Concentration	
Metals and metalloids	[24]	Portugal	Gouveia, Lisbon, Olhão	Hedgehog (*Erinaceus europaeus*)	Liver	Arsenic	0.14 ± 0.14	mg/kg dry weight
						Cadmium	0.95 ± 1.45	
						Nickel	0.04 ± 0.07	
						Lead	0.67 ± 1.12	
					Kidney	Arsenic	0.14 ± 0.15	
						Cadmium	3.50 ± 6.42	
						Nickel	0.24 ± 0.95	
						Lead	0.46 ± 0.95	
					Spines	Arsenic	0.22 ± 0.24	
						Cadmium	0.01 ± 0.01	
						Nickel	0.15 ± 0.21	
						Lead	0.48 ± 0.75	
	[25]	Portugal	Gouveia, Lisbon, Olhão	Hedgehog (*Erinaceus europaeus*)	Liver	Lead	0.54 ± 0.70	
						Cadmium	0.84 ± 1.27	
						Arsenic	0.13 ± 0.14	
	[22]	Portugal	Idanha-a-Nova	Red deer (*Cervus elaphus*)	Liver	Cadmium	0.39 ± 0.10	
						Lead	5.47 ± 6.78	
						Arsenic	0.05 ± 0.01	
					Kidney	Cadmium	5.86 ± 0.93	
						Lead	0.46 ± 0.58	
						Arsenic	0.08 ± 0.03	
			Lousã		Liver	Cadmium	0.32 ± 0.10	
						Lead	0.13 ± 0.07	
						Arsenic	0.04 ± 0.01	
					Kidney	Cadmium	13.06 ± 9.10	
						Lead	0.21 ± 0.10	
						Arsenic	0.13 ± 0.02	
	[23]	Spain	Castille and Léon	Wild boar (*Sus Scrofa*)	Liver	Nickel	0.08 ± 0.10	
						Lead	0.30 ± 0.40	
						Cadmium	0.70 ± 0.64	
						Arsenic	0.10 ± 0.05	
					Kidney	Nickel	0.25 ± 0.13	
						Lead	0.28 ± 0.17	
						Cadmium	7.06 ± 7.27	
						Arsenic	0.20 ± 0.09	
	[26]	Canada	Northern Beaufort Sea, Southern Hudson Bay, Western Hudson Bay, Baffin Bay, Foxe Basin, Gulf of Boothia	Polar bear (*Ursus maritimus*)	Muscle	Methylmercury	0.43 ± 0.33	
						Total mercury	0.59 ± 0.44	
						Lead	0.18 ± 0.42	
						Cadmium	0.07 ± 0.05	
						Arsenic	1.49 ± 1.55	
						Nickel	0.05 ± 0.06	
					Liver	Methylmercury	2.37 ± 2.50	
						Total mercury	45.86 ± 41.89	
						Lead	0.37 ± 0.55	
						Cadmium	2.07 ± 1.24	
						Arsenic	1.39 ± 1.32	
						Nickel	0.05 ± 0.07	
					Fat	Methylmercury	0.03 ± 0.03	
						Total mercury	5.55 ± 23.79	
						Lead	5.83 ± 39.98	
						Cadmium	0.04 ± 0.11	
						Arsenic	1.00 ± 0.79	
						Nickel	0.05 ± 0.05	
					Faeces	Methylmercury	0.31 ± 0.33	
						Total mercury	2.69 ± 3.52	
						Lead	449.55 ± 3144.01	
						Cadmium	1.59 ± 2.37	
						Arsenic	2.50 ± 3.29	
						Nickel	0.36 ± 0.69	
	[31]	Australia	Broken Hill	House sparrow (*Passer domesticus*)	Blood	Lead	31.4 ± 21.1	mg/dL
			Mount Isa				29.4 ± 22.8	
			Other *				11.0 ± 18.2	
	[29]	United States of America	Minesota	Opossum (*Didelphis virginiana*)	Blood	Lead	0.0147 ± 0.0197	
				Squirrel (*Sciurus carolinensis*)				
				Pigeon (*Columba livia*)			0.0067 ± 0.0126	

* Includes additional locations (Cobar, Dubbo, Cloncurry, Richmond, and Townsville) reported collectively in the original study.

**Table 2:** Pesticides accumulation levels (mean ± SD) detected across different species, sampling matrices, and geographic locations.

	Study	Country	Region	Species	Sample	Contaminant	Concentration	
Pesticides	[27]	China	Yunnan Province	Fulvous fruit bat (*Rousettus leschenaultia*)	Liver	Organochlorine insecticides	501.69 ± 216.83	µg/kg
				Great roundleaf bat (*Hipposideros armiger*)			821.65 ± 821.17	
				Chinese rufous horseshoe bat (*Rhinolophus sinicus*)			773.03 ± 589.29	
				Large myotis (*Myotis chinensis*)			627.62 ± 1176.30	
				Great evening bat (*Ia io*)			792.02 ± 1410.64	
				Eastern bet-wing bat (*Miniopterus fuliginosus*)			1167.78 ± 1063.66	
				Black bearded tomb bat (*Taphozous melanopogon*)			1176.38 ± 940.44	
				Fulvous fruit bat (*Rousettus leschenaultia*)		Organophosphorus insecticides	1149.19 ± 1648.08	
				Great roundleaf bat (*Hipposideros armiger*)			288.96 ± 171.17	
				Chinese rufous horseshoe bat (*Rhinolophus sinicus*)			192.92 ± 101.80	
				Large myotis (*Myotis chinensis*)			392.45 ± 117.91	
				Great evening bat (*Ia io*)			267.08 ± 103.15	
				Eastern bet-wing bat (*Miniopterus fuliginosus*)			331.05 ± 221.18	
				Black bearded tomb bat (*Taphozous melanopogon*)			263.81 ± 110.89	
				Fulvous fruit bat (*Rousettus leschenaultia*)		Carbamate insecticides	160.27 ± 182.07	
				Great roundleaf bat (*Hipposideros armiger*)			43.51 ± 38.18	
				Chinese rufous horseshoe bat (*Rhinolophus sinicus*)			40.92 ± 3.52	
				Large myotis (*Myotis chinensis*)			79.96 ± 61.99	
				Great evening bat (*Ia io*)			47.51 ± 20.38	
				Eastern bet-wing bat (*Miniopterus fuliginosus*)			42.07 ± 15.66	
				Black bearded tomb bat (*Taphozous melanopogon*)			40.46 ± 11.60	
				Fulvous fruit bat (*Rousettus leschenaultia*)		Pyrethroid insecticides	156.14 ± 192.06	
				Great roundleaf bat (*Hipposideros armiger*)			58.21 ± 41.16	
				Chinese rufous horseshoe bat (*Rhinolophus sinicus*)			37.69 ± 11.74	
				Large myotis (*Myotis chinensis*)			74.17 ± 81.63	
				Great evening bat (*Ia io*)			34.25 ± 4.56	
				Eastern bet-wing bat (*Miniopterus fuliginosus*)			63.51 ± 38.06	
				Black bearded tomb bat (*Taphozous melanopogon*)			48.02 ± 24.75	
				Fulvous fruit bat (*Rousettus leschenaultia*)		Acaricides	412.66 ± 754.38	
				Great roundleaf bat (*Hipposideros armiger*)			50.58 ± 91.25	
				Chinese rufous horseshoe bat (*Rhinolophus sinicus*)			9.48 ± 8.89	
				Large myotis (*Myotis chinensis*)			102.40 ± 99.37	
				Great evening bat (*Ia io*)			69.25 ± 26.97	
				Eastern bet-wing bat (*Miniopterus fuliginosus*)			59.02 ± 39.91	
				Black bearded tomb bat (*Taphozous melanopogon*)			56.07 ± 49.96	
				Fulvous fruit bat (*Rousettus leschenaultia*)		Herbicides	1962.71 ± 3719.72	
				Great roundleaf bat (*Hipposideros armiger*)			281.76 ± 196.80	
				Chinese rufous horseshoe bat (*Rhinolophus sinicus*)			149.34 ± 90.32	
				Large myotis (*Myotis chinensis*)			621.14 ± 531.69	
				Great evening bat (*Ia io*)			1057.05 ± 1533.88	
				Eastern bet-wing bat (*Miniopterus fuliginosus*)			260.12 ± 247.07	
				Black bearded tomb bat (*Taphozous melanopogon*)			298.69 ± 266.19	
				Fulvous fruit bat (*Rousettus leschenaultia*)		Fungicides	1297.33 ± 1965.89	
				Great roundleaf bat (*Hipposideros armiger*)			209.93 ± 267.51	
				Chinese rufous horseshoe bat (*Rhinolophus sinicus*)			80.41 ± 65.83	
				Large myotis (*Myotis chinensis*)			377.82 ± 239.05	
				Great evening bat (*Ia io*)			313.48 ± 42.56	
				Eastern bet-wing bat (*Miniopterus fuliginosus*)			251.76 ± 129.13	
				Black bearded tomb bat (*Taphozous melanopogon*)			283.10 ± 215.78	
				Fulvous fruit bat (*Rousettus leschenaultia*)		Others	123.17 ± 231.24	
				Great roundleaf bat (*Hipposideros armiger*)			27.06 ± 26.54	
				Chinese rufous horseshoe bat (*Rhinolophus sinicus*)			14.34 ± 8.39	
				Large myotis (*Myotis chinensis*)			74.12 ± 90.84	
				Great evening bat (*Ia io*)			44.55 ± 20.45	
				Eastern bet-wing bat (*Miniopterus fuliginosus*)			25.07 ± 29.24	
				Black bearded tomb bat (*Taphozous melanopogon*)			25.44 ± 15.69	
	[28]	United States of America	Michigan	Skunk (*Mephitis mephitis*)	Brain	Organochlorine insecticides (chlordane and metabolites *)	4500 ± 7120	ng/g wet weight
							101,000 ± 110,000	ng/g lipid weight
					Liver		87,200 ± 201,000	ng/g wet weight
							697,000 ± 1,030,000	ng/g lipid weight

* Chlordane metabolites include oxychlordane, heptachlor epoxide, and trans-nonachlor.

**Table 3:** Microplastics accumulation levels (mean ± SD) detected in regurgitated pellets from different raptor species.

	Study	Country	Region	Species	Sample	Contaminant	Concentration	
Microplastics	[32]	Spain	Almería, Granada, Cádiz, Córdoba, Jaén, Mallorca, Málaga, Madrid, Ávila, Burgos, La Rioja, Salamanca, Cáceres, Zamora, Badajos	Cinereous vulture (*Aegypius monachus*)	Regurgitated pellet	AFs	4.27 ± 1.23	AFs/pellet
				Bonelli’s eagle (*Aquila fasciata*)			7.60 ± 1.21	
				Little owl (*Athene noctua*)			0.88 ± 0.48	
				Lesser kestrel (*Falco naumanni*)			2.60 ± 0.81	
				Red kite (*Milvus milvus*)			4.12 ± 1.74	
				Barn owl (*Tyto alba*)			7.90 ± 3.97	
				Cinereous vulture (*Aegypius monachus*)		MPs	3.90 ± 1.56	MPs/pellet
				Bonelli’s eagle (*Aquila fasciata*)			2.74 ± 0.78	
				Little owl (*Athene noctua*)			1.59 ± 0.54	
				Lesser kestrel (*Falco naumanni*)			0.85 ± 0.46	
				Red kite (*Milvus milvus*)			4.21 ± 0.95	
				Barn owl (*Tyto alba*)			2.17 ± 0.70	

**Table 4:** Zearalenone (ZEN) and α-zearalenol (α-ZEL) accumulation levels (mean ± SD) detected in the liver, muscle and kidneys of wild boar (*Sus scrofa*).

	Study	Country	Region	Species	Sample	Contaminant	Concentration	
Mycotoxins	[15]	Italy	Avellino Province	Wild Boar (*Sus Scrofa*)	Liver	ZEN	1.71 ± 1.98	ng/g
					Muscle		1.49 ± 2.26	
					Kidney		0.65 ± 0.90	
					Liver	α-ZEL	0.65 ± 0.96	
					Muscle		0.66 ± 0.57	
					Kidney		0.77 ± 0.98	

## Data Availability

No new data were created or analyzed in this study.

## Abbreviations

The following abbreviations are used in this manuscript:

SD	Standard deviation
SE	Standard error of the mean
ZEN	Zearalenone
α-ZEL	α-zearalenol
AFs	Artificial fibers
MPs	Microparticles

## References

[1] Usman G., Mashood A.A., Aliyu A., Adamu K.S., Salisu A., Abdullahi A.K., Sheriff H.K. (2023). Effects of environmental pollution on wildlife and human Health and novel mitigation strategies. World J. Adv. Res. Rev..

[2] Destoumieux-Garzon D., Mavingui P., Boetsch G., Boissier J., Darriet F., Duboz P., Fritsch C., Giraudoux P., Le Roux F., Morand S. (2018). The One Health Concept: 10 Years Old and a Long Road Ahead. Front. Vet. Sci..

[3] Schwarzenbach R.P., Egli T., Hofstetter T.B., von Gunten U., Wehrli B. (2010). Global Water Pollution and Human Health. Annu. Rev. Environ. Resour..

[4] Daley J.M., Paterson G., Drouillard K.G. (2014). Bioamplification as a bioaccumulation mechanism for persistent organic pollutants (POPs) in wildlife. Rev. Environ. Contam. Toxicol..

[5] Taggart M.A., Reglero M.M., Camarero P.R., Mateo R. (2011). Should legislation regarding maximum Pb and Cd levels in human food also cover large game meat?. Environ. Int..

[6] Ali H., Khan E., Ilahi I. (2019). Environmental Chemistry and Ecotoxicology of Hazardous Heavy Metals: Environmental Persistence, Toxicity, and Bioaccumulation. J. Chem..

[7] Seabert T.A., Pal S., Pinet B.M., Haman F., Robidoux M.A., Imbeault P., Krummel E.M., Kimpe L.E., Blais J.M. (2014). Elevated contaminants contrasted with potential benefits of omega-3 fatty acids in wild food consumers of two remote first nations communities in northern Ontario, Canada. PLoS ONE.

[8] Moriarity R.J., Tsuji L.J.S., Liberda E.N. (2023). A probabilistic hazard and risk assessment of exposure to metals and organohalogens associated with a traditional diet in the Indigenous communities of Eeyou Istchee (northern Quebec, Canada). Environ. Sci. Pollut. Res. Int..

[9] Nkosi D.V., Bekker J.L., Hoffman L.C. (2021). Toxic Metals in Wild Ungulates and Domestic Meat Animals Slaughtered for Food Purposes: A Systemic Review. Foods.

[10] Jomova K., Alomar S.Y., Nepovimova E., Kuca K., Valko M. (2025). Heavy metals: Toxicity and human health effects. Arch. Toxicol..

[11] Danieli P.P., Serrani F., Primi R., Ponzetta M.P., Ronchi B., Amici A. (2012). Cadmium, lead, and chromium in large game: A local-scale exposure assessment for hunters consuming meat and liver of wild boar. Arch. Environ. Contam. Toxicol..

[12] A Health Perspective on the Role of the Environment in One Health. https://apps.who.int/iris/handle/10665/352919.

[13] Warenik-Bany M., Strucinski P., Piskorska-Pliszczynska J. (2016). Dioxins and PCBs in game animals: Interspecies comparison and related consumer exposure. Environ. Int..

[14] Cverenkarova K., Valachovicova M., Mackulak T., Zemlicka L., Birosova L. (2021). Microplastics in the Food Chain. Life.

[15] Longobardi C., Damiano S., Ferrara G., Montagnaro S., Meucci V., Intorre L., Bacci S., Esposito L., Piscopo N., Rubino A. (2023). Zearalenone (ZEN) and Its Metabolite Levels in Tissues of Wild Boar (*Sus scrofa*) from Southern Italy: A Pilot Study. Toxins.

[16] Kaczynski P., Lozowicka B., Perkowski M., Zon W., Hrynko I., Rutkowska E., Skibko Z. (2021). Impact of broad-spectrum pesticides used in the agricultural and forestry sector on the pesticide profile in wild boar, roe deer and deer and risk assessment for venison consumers. Sci. Total Environ..

[17] Wang F., Xiang L., Sze-Yin Leung K., Elsner M., Zhang Y., Guo Y., Pan B., Sun H., An T., Ying G. (2024). Emerging contaminants: A One Health perspective. Innovation.

[18] Needham T., Bureš D., Černý J., Hoffman L.C. (2023). Overview of game meat utilisation challenges and opportunities: A European perspective. Meat Sci..

[19] Ramanzin M., Amici A., Casoli C., Esposito L., Lupi P., Marsico G., Mattiello S., Olivieri O., Ponzetta M.P., Russo C. (2010). Meat from wild ungulates: Ensuring quality and hygiene of an increasing resource. Ital. J. Anim. Sci..

[20] AlRyalat S.A.S., Malkawi L.W., Momani S.M. (2019). Comparing Bibliometric Analysis Using PubMed, Scopus, and Web of Science Databases. J. Vis. Exp..

[21] Wan X., Wang W., Liu J., Tong T. (2014). Estimating the sample mean and standard deviation from the sample size, median, range and/or interquartile range. BMC Med. Res. Methodol..

[22] Baptista C.J., Seixas F., Gonzalo-Orden J.M., Patinha C., Pato PFerreira da Silva E., Fernandes G., Oliveira P.A. (2024). Heavy metal and metalloid concentrations in red deer (*Cervus elaphus*) and their human health implications from One Health perspective. Environ. Geochem. Health.

[23] Baptista C.J., Seixas F., Gonzalo-Orden J.M., Patinha C., Pato P., Ferreira da Silva E., Merino-Goyenechea L.J., Oliveira P.A. (2024). Heavy metals and metalloids in wild boars (*Sus Scrofa*)—A silent but serious public health hazard. Vet. Res. Commun..

[24] Baptista C.J., Seixas F., Gonzalo-Orden J.M., Patinha C., Pato P., Ferreira da Silva E., Casero M., Brazio E., Brandao R., Costa D. (2024). The first full study of heavy metal(loid)s in western-European hedgehogs (*Erinaceus europaeus*) from Portugal. Environ. Sci. Pollut. Res. Int..

[25] Baptista C.J., Seixas F., Gonzalo-Orden J.M., Patinha C., Pato P., Ferreira da Silva E., Casero M., Brazio E., Brandao R., Costa D. (2023). High Levels of Heavy Metal(loid)s Related to Biliary Hyperplasia in Hedgehogs (*Erinaceus europaeus*). Animals.

[26] Eccles K.M., Boutet V., Branigan M., Dyck M., van Coeverden de Groot P., Lougheed S.C., Rutter A., Langlois V.S. (2024). Non-invasive biomonitoring of polar bear feces can be used to estimate concentrations of metals of concern in traditional food. PLoS ONE.

[27] Wang Y., Huang X., Zhang X., Yang J., Liu Y., Ke C., Taylor P.J., Feng J., Jiang T. (2026). Occurrence and determinants of multi-pesticide residues in bats: A case study in Yunnan Province, China. Environ. Pollut..

[28] Sheffler R., Puschner B., Melotti J., Fitzgerald S.D., Buchweitz J.P. (2025). Chlordane-Induced Neurotoxicosis in Urban and Suburban Detroit, Michigan Striped Skunks (*Mephitis mephitis*). Toxics.

[29] Imagawa M., Rushing M., Carter A., Schott R., Berman J.D. (2023). Using blood lead concentrations of wildlife sentinels to identify environmental risk factors of lead exposure for public health and wildlife rehabilitation efforts. Ecotoxicology.

[30] Kalani T.J., South A., Talmadge C., Leibler J., Whittier C., Rosenbaum M. (2021). One map: Using geospatial analysis to understand lead exposure across humans, animals, and the environment in an urban US city. One Health.

[31] Gillings M.M., Ton R., Harris T., Swaddle J.P., Taylor M.P., Griffith S.C. (2024). House Sparrows as Sentinels of Childhood Lead Exposure. Environ. Sci. Technol..

[32] Wayman C., Fernández-Piñas F., López-Márquez I., Fernández-Valeriano R., Iglesias-Lebrija J.J., González-González F., Rosal R., González-Pleiter M. (2024). Unraveling Plastic Pollution in Protected Terrestrial Raptors Using Regurgitated Pellets. Microplastics.

[33] Underwood E.J. (1981). The incidence of trace element deficiency diseases. Philos. Trans. R. Soc. Lond. Ser. B Biol. Sci..

[34] Rosa S., Silvestre-Ferreira A.C., Queiroga F.P. (2025). A Review of the Sentinel Role of *Erinaceus europaeus* in Zoonotic Diseases Across Urban and Rural Environments: A One Health Perspective. Vet. Sci..

[35] Dominique M., Letcher R.J., Rutter A., Langlois V.S. (2020). Comparative review of the distribution and burden of contaminants in the body of polar bears. Environ. Sci. Pollut. Res. Int..

[36] Thomas V.G., Pain D.J., Kanstrup N., Cromie R. (2022). Increasing the Awareness of Health Risks from Lead-Contaminated Game Meat Among International and National Human Health Organizations. Eur. J. Environ. Public Health.

[37] Mandal G.C., Mandal A., Chakraborty A. (2023). The toxic effect of lead on human health. Hum. Biol. Public Health.

[38] Ciobanu M.M., Boisteanu P.C., Munteanu M., Târziu D., Ratu R.N., Postolache A.N. (2021). Bioavailability of heavy metals (Pb and Cd) in wild roe deer meat. Sci. Pap. Ser. D Anim. Sci..

[39] Binkowski Ł.J., Kalisińska E. (2019). Arsenic, As. Mammals and Birds as Bioindicators of Trace Element Contaminations in Terrestrial Environments.

[40] Katzner T.E., Pain D.J., McTee M., Brown L., Cuadros S., Pokras M., Slabe V.A., Watson R.T., Wiemeyer G., Bedrosian B. (2024). Lead poisoning of raptors: State of the science and cross-discipline mitigation options for a global problem. Biol. Rev. Camb. Philos. Soc..

[41] Burger J. (2008). Assessment and management of risk to wildlife from cadmium. Sci. Total Environ..

[42] Charkiewicz A.E., Omeljaniuk W.J., Nowak K., Garley M., Niklinski J. (2023). Cadmium Toxicity and Health Effects-A Brief Summary. Molecules.

[43] Hong Y.S., Song K.H., Chung J.Y. (2014). Health effects of chronic arsenic exposure. J. Prev. Med. Public Health.

[44] Wolfe M.F., Schwarzbach S., Sulaiman R.A. (1998). Effects of mercury on wildlife: A comprehensive review. Environ. Toxicol. Chem..

[45] Park J.D., Zheng W. (2012). Human exposure and health effects of inorganic and elemental mercury. J. Prev. Med. Public Health.

[46] Das K.K., Das S.N., Dhundasi S.A. (2008). Nickel, its adverse health effects & oxidative stress. Indian J. Med. Res..

[47] Berny P. (2007). Pesticides and the intoxication of wild animals. J. Vet. Pharmacol. Ther..

[48] Krief S., Spirhanzlova P., Masi S., Couturier C., Okwir E., Asalu E., Bustamante P., Costantini D. (2023). High urinary oxidative DNA damage in wild chimpanzees ranging in proximity of agricultural fields in Sebitoli area, Uganda. Environ. Sci. Pollut. Res. Int..

[49] Leemans M., Couderq S., Demeneix B., Fini J.B. (2019). Pesticides with Potential Thyroid Hormone-Disrupting Effects: A Review of Recent Data. Front. Endocrinol..

[50] Laskar N., Kumar U. (2019). Plastics and microplastics: A threat to environment. Environ. Technol. Innov..

[51] da Rocha M.E.B., Freire F.d.C.O., Maia F.E.F., Guedes M.I.F., Rondina D. (2014). Mycotoxins and their effects on human and animal health. Food Control.

